# Correspondence

**Published:** 1874-08

**Authors:** G. D. Hodge

**Affiliations:** Holly Springs, Arkansas


### CORRESPONDENCE.

### *A Very Simple, but Efficient Apparatus in the Treatment of Fractured Clavicle.*

From the multiplicity of contrivances from time to time presented for the treatment of clavicular fracture, it may seem entirely superfluous to advance anything which shall claim to do more than has, long since, been done. This we do not at all attempt; it is only the *manner* in which it shall be accomplished that is simplified by the plan we have to describe.

It is unnecessary to repeat the indications for treatment, or recapitulate the various methods for their carrying out, in fractures of this bone. One thing is *certain*, their *perfect* fulfillment is *impossible.* No apparatus ever yet devised has accomplished it, and, according to the highest authority on fractures in this country, Prof. Frank Hamilton, "the third indication to carry the shoulder out, still remains unaccomplished, * * * * * nor have I much confidence that this end, so desirable, and so diligently sought, will ever be attained."

Since then, the most to be hoped for is to reduce the deformity to the highest *minimum* amount, and do this with the greatest comfort and safety to the patient. Any device answering this purpose cannot be without some features to commend it.

To accomplish this, we believe the recumbent posture, with the head low, and a pillow or other pad between the shoulders so as to allow them to fall outwards and backwards by their own weight, will yield results more satisfactory than any other plan whatever, but it is very exceptionally that a patient can be found who will submit to the long confinement in bed, and we are then compelled to employ some other form of treatment.

In this country and Great Britain, no other plan is so generally adopted as that of Fox, either as originally introduced, or with the modifications as recommended. After reviewing various methods, Prof. Hamilton reaches the conclusion that this is the one to be preferred, with the improvements suggested by himself.

Many surgeons employ adhesive plaster, confining the arm to the body, and elevating the humeral portion of the broken bone, by a broad strip passed around the elbow and over the shoulder of the opposite side. This plan stands first on the list of appliances recommended by Professor Gross, and Professor Sayre adopts one very similar, the only difference being the crossing of the arm behind instead of in front.

With some, probably the majority, this procedure will answer every purpose; but there are those on whom adhesive plaster produces an erysipelatous inflammation, and it cannot be tolerated. Again, in warm weather, it is sometimes excessively uncomfortable, and if it chance to be of inferior quality, scarcely anything will prevent it slipping, and thereby defeating the very object most desired.

The simple plan we have to present, which obviates all these objections, is as follows: a piece of unyielding leather, cup-shaped, to fit the elbow, to the outside of which is to be attached a strip of ordinary webbing, one and one-half inches wide, such as is used for driving reins, provided with a buckle at one end, and a leather strap one-half inch in width, and five or six inches long at the other, the whole of sufficient length to reach from the elbow of the affected side, passing beneath the scapula of the same, over the opposite shoulder, and return. The forearm should be placed across the chest, a little above a right angle with the humerus, and an ordinary surgical bandage fastened to the wrist, be passed over the fractured point, and properly secured to the webbing behind.

If the piece fitted to the elbow be fastened about fifteen inches from the end to which the buckle is attached, the ends will meet opposite one or the other breast, and be more easily adapted than elsewhere. The elbow-cup is to be well lined with cotton, and the same interposed wherever the aparatus will be liable to press unduly, and, if desired, a firm pad can be placed under the shoulder-strap, so as to make the pressure direct upon the point of fracture.

This completes the dressing. No axillary pad; no fastening around the body, and no extra straps or bandages. It is light, comfortable and convenient. All that is required in its adjustment is to place the arm in position and buckle up the main strap, thereby elevating the shoulder, until the opposite fragments of the broken bone are directly in line. That it will accomplish its object, after the failure of other methods, the following case will attest:

SEPTEMBER 12, 1872.—A. T., æt. 19, by a railroad collision, was thrown twenty feet or more in the air, alighting on his head and shoulders. He was taken up unconscious, and an examination disclosed an oblique fracture of the clavicle, at the outer end of the middle third. Patient was ordered to bed, head low, and pillows between the shoulders. Forty-eight hours subsequently, pain in the head having subsided, and confinement in bed being tiresome, he refused to remain longer, and the adhesive plaster dressing, as recommended by Prof. Gross, was applied. Two days after it was loose; was reapplied, and over it the ordinary surgical roller. Three days later, erysipelatous inflammation showed itself at various points in the track of the plaster, and the patient making much complaint of this and the close dressing, it had to be removed. We then employed the appliance above described, which proved perfectly comfortable, and answered every indication, save, of course, the *complete* carrying the shoulder out. At the expiration of the ordinary time, patient was discharged with ünion firm and direct, and no deformity, other than a very slight overlapping.

We claim nothing particularly *new* in the dressing. It is merely a variety of the ordinary sling arrangement; but it is simple and effectual, and possesses an advantage over the handkerchief, simplest of all appliances, in being more easily managed and kept in place, as to commend it somewhat to the attention of practitioners, in the management of fractured clavicle. *

TANSY.—Some time since we saw—I believe in the RECORD—a casual notice of the common garden tansy, as a remedy for epistaxis. We chanced to read the article in the hearing of the family, and suggested to Alice a trial of its efficacy, the first time little Charlie should come in with a bleeding proboscis. Within a very few days, "young America" was ushered in, with the blood streaming from a smashed-up nose. Of course, no time was lost in applying the bruised herb, in the shape of a plug, and the hemorrhage ceased *almost instanter*. There being a full school of little chaps hard by, with their play ground near the garden, case after case soon came in, and was treated with like success; and tansy at once arose in the estimation of our matrons as a *specific*. We will also add that we have, time and again, seen the bleeding speedily arrested by merely inhaling, or rather *snuffing*, up the nostril the pungent odor of a bruised tansy boquet, thereby favoring the idea that the remedy exercises some *therapeutical*, as well as mechanical, agency. But *this* is not *all*. A few days since it was our lot to treat a case of alarming abortion in the person of a colored patient, in destitute circumstances. On arrival, soon found the matter stood, *tampon or death*. Now, don't smile—if you *publish*, I will not read *this* to the family. A huge bowl of "double tansy," from which patient had been drinking the tea, was sitting on the hearth, and finding no suitable fabric at hand, we gathered up the infused herb and *literally crammed* the vagina, with enough protruding to suggest to one's mind the idea of a bunch of mistletoe. In less than thirty minutes the patient rallied; os dilated, and the foetus was expelled, preceded by a mass of firmly coagulated blood. Now, admitting that the plug acted *mechanically* only, and I must say, under like circumstances, we would not hesitate to construct our tampon of *tansy*. Try it, any way, for epistaxis. G. D. HODGE, M.D.

HOLLY SPRINGS, ARKANSAS, May 22, 1874.

